# The Advanced Integrated Respiratory (AIR) Model: Integration of Air–Liquid Interface Cell Cultures within a Human Airway Model for Inhalation Toxicology

**DOI:** 10.1007/s11095-026-04037-z

**Published:** 2026-02-25

**Authors:** Patrick He, Hanieh Gholizadeh, Damien Chong, Shaokoon Cheng, Patrick Spicer, Paul Michael Young, Lois Ledo, Vanessa Wilson, Daniela Traini, Hui Xin Ong

**Affiliations:** 1 Respiratory Technology, Woolcock Institute of Medical Research, Macquarie University, Sydney, NSW Australia; 2 Macquarie Medical School, Faculty of Medicine, Health and Human Sciences, Macquarie University, Sydney, NSW Australia; 3 DSTG, Fishermans Bend, VIC, Australia; 4 School of Chemical Engineering, University of New South Wales, Kensington, NSW Australia; 5 Macquarie Business School, Macquarie University, Sydney, NSW Australia; 6 School of Engineering, Macquarie University, Sydney, Australia

**Keywords:** *in vitro* model, MAD, pulmonary delivery, RCA I, respiratory aerosols

## Abstract

**Purpose:**

The Advanced Integrated Respiratory (AIR) model was developed as a physiologically relevant benchtop system designed to assess aerosol deposition and interactions within the respiratory tract.

**Methods:**

This model integrates a three-dimensional (3D) cast of the human airways with a vacuum driven aerosol inhalation flow and an air liquid interface (ALI) cell culture platform. In this study, the integrated AIR and ALI cell model was used to investigate the toxicity profile of aerosolized Ricinus communis agglutinin-1 (RCA I) toxin. RCA I was characterized in terms of particle size, surface charge, rheology, and aerosol performance. Additionally, real-time electrochemical detection using the Micro Analytical Device (MAD) provided high sensitivity quantification of aerosolized RCA I. The biological effects were assessed using human epithelial cells cultured under ALI conditions, which were exposed to RCA I aerosols. Cytotoxicity and barrier function assays were performed to evaluate its impact.

**Results:**

Results show significant differences in toxic dose thresholds comparing 2D and AIR models. Transport study revealed that RCA I exhibited significantly increased mass transport across the epithelial cell layer at toxic concentrations compared to non-toxic concentrations.

**Conclusions:**

This integrated approach represents a significant advancement in the study of inhaled aerosol deposition, toxicity, and pharmacokinetics, offering a robust tool for predicting lung injury and enhancing the detection of a wide range of inhaled aerosols, including but not limited to toxins.

## Introduction

Accurate *in vitro* models that replicate human respiratory physiology are critical for advancing research in aerosolized drug delivery, inhalation toxicology, and the characterization of airborne toxins. Three-dimensional (3D) printed respiratory models have gained popularity for studying particle deposition in the respiratory tract, addressing the limitations of current *in vitro* and *in vivo* models [1]. *In vivo* animal models provide insight into aerosol absorption and pharmacodynamics but pose ethical, maintenance, and interspecies variability concerns [2]. Traditionally *In vitro* two-dimensional (2D) cell models have been used to advance our understanding of drug toxicology and disease mechanisms, however, their inability to reproduce key features of the respiratory epithelium such as mucous production, multicellular organization, and physiologically relevant geometry, creates gaps in mechanistic interpretation [2, 3]. This has driven the development of more complex 3D platforms, including respiratory organoids, lung on chip systems, air liquid interface (ALI) cultures, and mouth to airway models such as the Andersen Cascade Impactor (ACI) and Next Generation Impactor (NGI), at the forefront of preclinical screening [4].

Despite these advancements, current respiratory models remain limited in their ability to replicate human inhalation physiology [3]. Organoid integrated with lung on chip systems are able to simulate *in vivo* breathing mechanics in physiologically representative microenvironments, however, they lack anatomical accurate airway geometry and capacity to carry out aerosol deposition studies [4]. Commercial ALI platforms such as Vitrocell® and Cultex® provide direct exposure of cell cultures to gasses, aerosols, and complex mixtures, like organoids, they cannot model regional deposition or the anatomical airway geometry [5, 6]. In contrast, ACI and NGI models can characterise aerodynamic particle size distribution and regional deposition, but they do not incorporate biological microenvironment or breathing mechanics [4].

The Advanced Integrated Respiratory (AIR) Model addresses the limitation of *in vitro *models by integrating biological relevance with anatomical fidelity to present a single physiologically relevant bench top system. This model incorporates a Magnetic Resonance Imaging (MRI)-derived 3D cast of the upper and lower airways, allowing physiologically accurate airflow patterns and region-specific aerosol deposition. An integrated vacuum system facilitates the transport of aerosols through the airway, while a specialised filtration system ensures containment of the aerosolized agents which more closely mimics inhalation compared with static exposure chambers such as organoids and 2D cell cultures [7]. AIR model has the capacity to incorporate ALI cultures of human respiratory epithelial cells, enabling a more physiologically relevant investigation of aerosol-cell interactions [8]. Unlike Vitrocell® [5] and Cultex® [6], which deliver aerosols to static ALI cultures within simplified chamber geometries, the AIR model integrates these ALI cultures directly within anatomically representative airway architecture. This capability is particularly important for assessing the deposition and biological effects of inhaled agents, allowing for a more accurate prediction of *in vivo* responses which current *in vitro* models cannot achieve [4]. In conjunction with the AIR model, the MAD was developed as a high-sensitivity analytical tool for the quantification of aerosolized toxins, chemicals, and nanoparticles. Utilising advanced detection techniques, the MAD offers superior sensitivity compared to conventional methods such as high-performance liquid chromatography (HPLC), facilitating precise measurement of aerosol exposure at low concentrations.

In this study, the AIR model is employed to investigate the deposition and toxicity profiles of aerosolized Ricinus communis agglutinin-1 (RCA I), a homologous but significantly less toxic analog of ricin [9]. RCA I was selected as a representative protein for assessing inhalation exposure risks due to its well-characterized physicochemical properties and biological activity [10]. The study encompasses a comprehensive characterization of RCA I, including its aerosol performance, rheology, and electrochemical detection via the MAD system. Additionally, cytotoxicity and barrier function assays were conducted using human distal lung epithelial cells (H441) cultured under ALI conditions to evaluate RCA I’s impact on epithelial integrity [11]. Through integration with the AIR model, the deposition and toxicity of RCA I aerosols were systematically assessed, demonstrating the utility of this platform in modelling real-world inhalation exposure scenarios.

## Material and Methods

### Materials

RCA I was purchased from Vector Laboratories, USA and Hanks’ Balanced Salt Solution (HBSS) from Sigma Aldrich (Missouri, USA). Unless mentioned otherwise, all other chemicals used in this study were purchased from Chem-Supply Pty Ltd. (Adelaide, Australia). Water was purified by reverse osmosis (MilliQ, Millipore, Guyancourt, France). All solvents used were of analytical grade.

### Methods

#### Development of the AIR Model

**Fig. 1:**
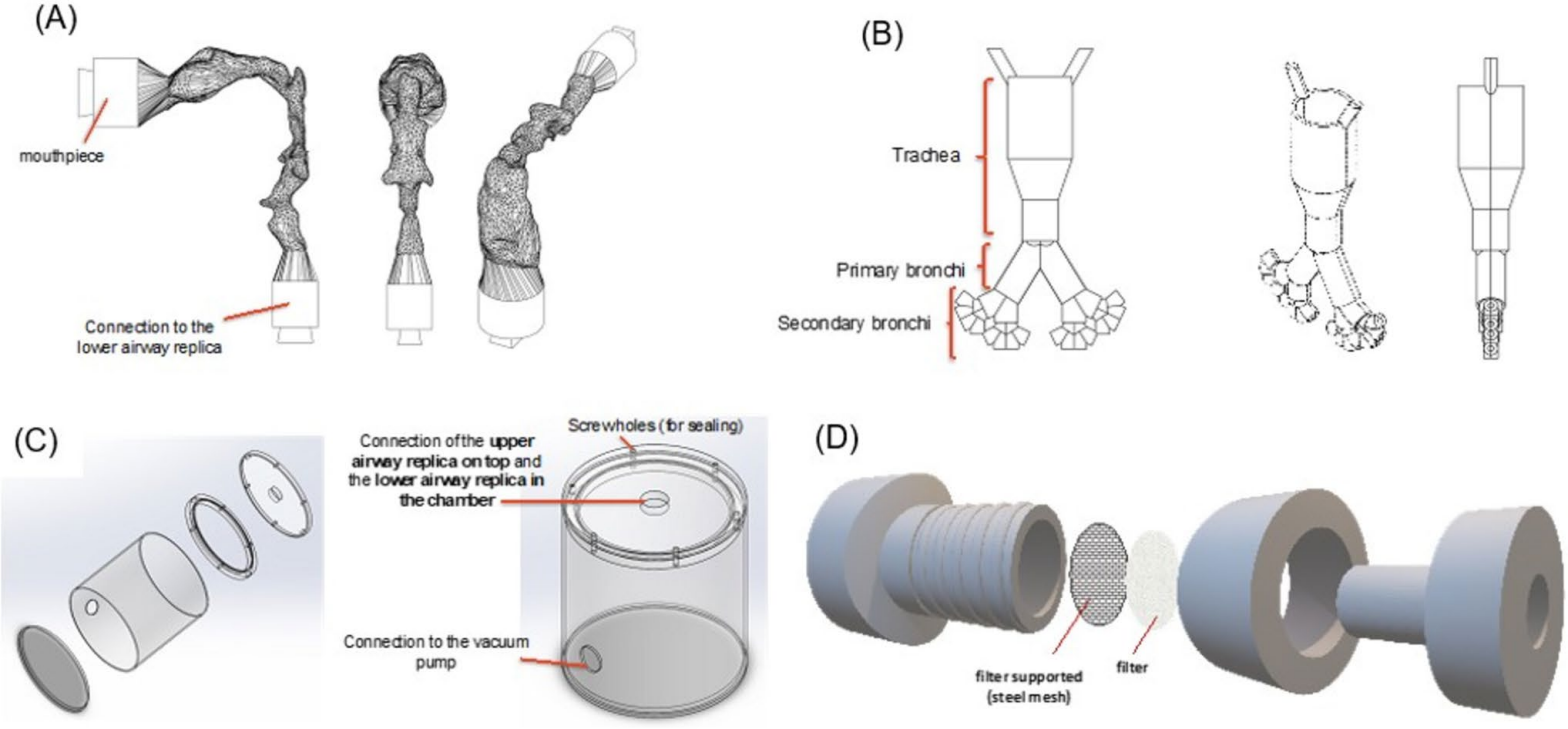
Main components of the AIR model: (**A**) the silicone upper airway model, (**B**) the silicone lower airway model, (**C**) the plexiglass vacuum chamber, and (**D**) filter and gauze at the connection to the vacuum pump.

The AIR model consists of an upper airway component (including the oropharynx, uvula, epiglottis, and soft palate; Fig. 1A) reconstructed from anatomical images acquired via MRI of an average, healthy adult male volunteer. This is combined with four generations of the Weibel lower airway model [12], beginning at the trachea (generation 0) and extending to the subsegmental bronchi (generation 4) (Fig. 1B) [13, 14]. Larger aerosol particles (> 5 µm) will predominantly deposit within the first 4 generations due to inertia impaction, whereas smaller particles (< 1–5 µm) in the distal bronchioles becomes less dependent on detailed geometric anatomy and more influenced by diffusion and gravitational sedimentation [15]. Extending the physical model beyond generation 4 would significantly increase fabrication complexity without providing proportional gains in predictive accuracy [15, 16]. Hence, to captured distal airway behaviour in the AIR model, we integrated ALI cell inserts at the terminal ends of generation 4, enabling a simplified yet physiologically relevant representation of deposition and cellular interaction. The complete airway model is fabricated using a 3D-printed mould, which is then inversely cast with optically transparent silicone. The silicone lower airway model is placed within a plexiglass vacuum chamber (20 cm diameter × 27 cm height), with an internal volume of approximately 8.5 L, that functions as a surrogate lung environment. This volume was selected to approximate the average adult male total lung capacity (~ 6 L), while providing additional space to accommodate model components [17]. The chamber’s diameter also reflects the approximate transverse width of the lungs in the anterior view [18]. This setup confines aerosols exiting the lower airways, thereby functionally mimicking the pulmonary cavity (Fig. 1C and D).

#### Assembly of the AIR Model

A vacuum chamber was connected to a vacuum pump (Westech Scientific Instrument, Chelmsford, UK) equipped with a microfiber filter (Sigma Aldrich, Missouri, United States) at the outlet to capture aerosols that may leave the chamber through the vacuum port. The vacuum chamber lid was then connected with the lower airway and screwed onto the vacuum chamber. Then, the upper airway was connected to the top of the vacuum chamber lid, and the mouthpiece was connected to the upper airway. The mouthpiece was held by a grip stand, and the position of the vacuum chamber was adjusted properly to avoid any twist in the upper airway. The AIR model operates under steady-state inhalation flow, controlled with a Copley flow controller (Nottingham, UK) and checked using a calibrated TSI 4040 flow meter connected to the mouthpiece. The airflow was set to 15 L/min with the vacuum pump running. All tests were carried out under normal laboratory conditions. Experimental tests were performed under ambient laboratory conditions.

#### Characterization of Aerosol Deposition in the AIR Model using Flu-NA Aerosols

**Fig. 2:**
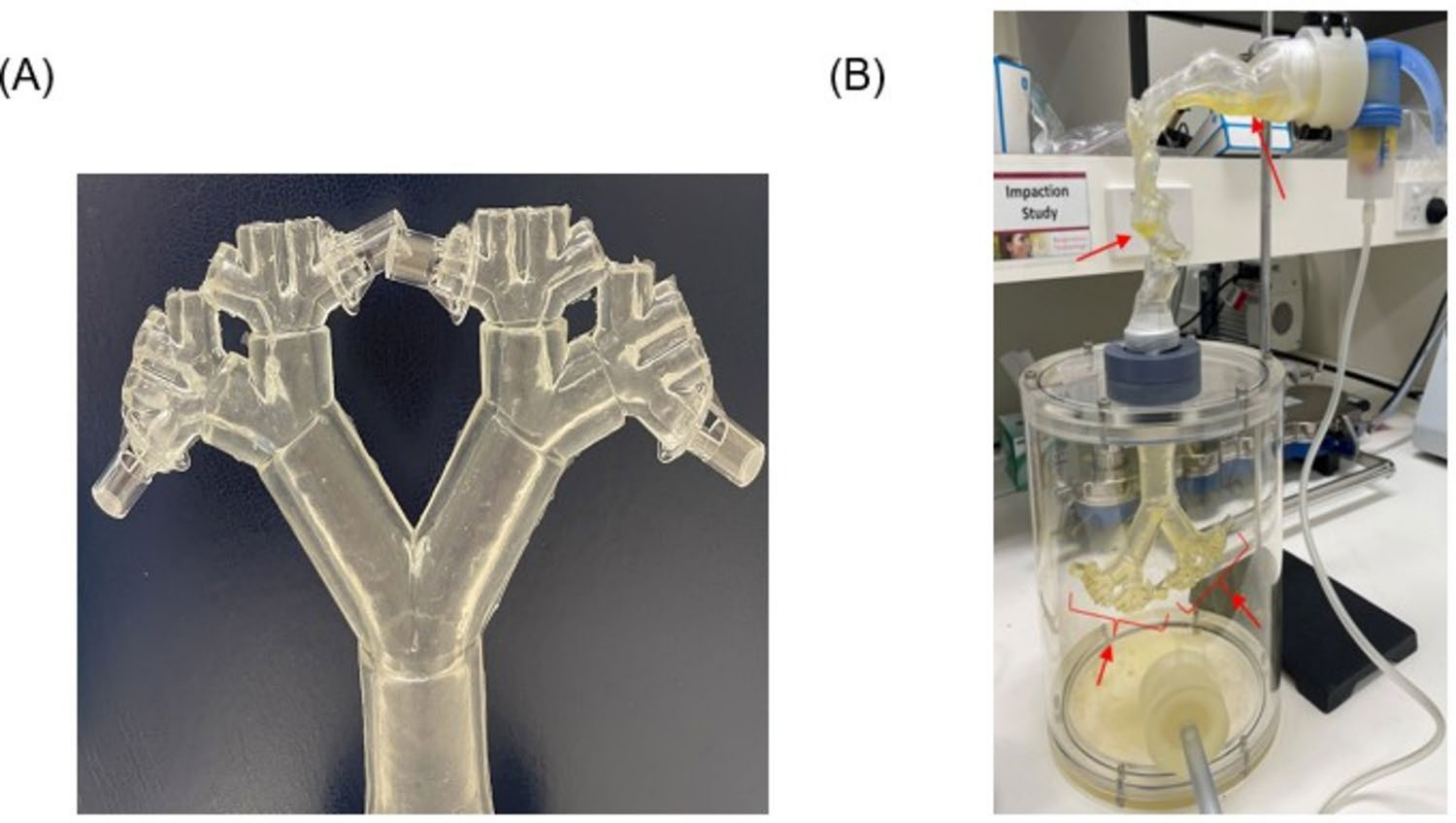
The experimental set-up of the integrated AIR model with ALI Transwell inserts. (**A**) connection of the Transwell inserts to the lower airway. (**B**) complete experimental setup for simulating aerosol delivery by the AIR model. The annotated red arrows represent the highest observed deposition of Flu-Na dye solution nebulized in the AIR model at 15 L/min flow rate.

Sodium Fluorescein (Flu-Na) was used as a model aerosol to characterise the AIR model and determine mass aerosol deposition. Flu-Na was aerosolized under three setups, without Transwells, with four empty Transwells, and with four H441 cultured Transwells connected to the lower airway (Fig. 2A) AIR model. Transwells positioned at the terminal end of the AIR model were used to capture and evaluate aerosol deposition in the distal lung region. These inserts were seeded with H441 cells, a human pulmonary epithelial cell line derived from the distal airway, to enable functional assessment of aerosol delivery at the bronchiolar–alveolar interface.1 mL Flu-Na solution in PBS (2.5 mg/mL) was transferred to a PARI LC® Sprint jet nebuliser and the solution was aerosolized into the AIR model at 15 L/min for 2 min, as shown in Fig. 2B.

Each AIR model component was placed in a suitably sized beaker and rinsed with Milli-Q water for Flu-Na quantification. Samples were transferred into a black 96-well plate for analysis. Fluorescence intensity was measured at 485 nm (excitation) and 583 nm (emission) using a SpectraMax M2 plate reader (Molecular Devices, California, USA). A calibration curve (R^2^ = 0.999) was obtained using standards with concentrations from 0.0125 to 1.25 μg/mL.

#### Aerosol Size Characterization of RCA I

The next generation impactor (NGI, Copley Scientific, Nottingham,UK) was used to study the delivery of the aerosolized RCA I solutions to different regions of the lungs based on the aerodynamic diameter of the aerosol particles. The airflow was set at 15 L/min by using a vacuum pump (Westech Scientific Instruments, Chelmsford, UK) and calibrated using a Mass Flow Meter 4040 (TSI Precision Measurement Instruments, Aachen, Germany).

RCA I (0.6 mg/mL) solutions were prepared in HBSS. 4 mL solution of RCA I was added to a PARI LC® Sprint jet nebuliser connected to a PARI Turbo Boy S compressor (Starnberg, Germany) to generate inhalable aerosols. Each stage of the NGI was thoroughly washed with milli-Q water (5 mL) in addition to the throat (3 mL), mouthpiece (3 mL), and the nebuliser device (10 mL) to determine the amount of RCA I deposited on the NGI. The collected samples were transferred into appropriate volumetric flasks for analytical analysis by high performance liquid chromatography (HPLC) using a validated method. Experimental tests were performed under ambient laboratory conditions.

#### Quantification of RCA I by High Performance Liquid Chromatography

RCA I content was assessed using an HPLC system equipped with SPD-20A UV–VIS detector, a LC-20AT liquid chromatography, a SIL-20A HT sampler (Shimadzu, Kyoto, Japan) and a Waters Luna C18 100 Å, Column (2.7 μm, 50 × 2.1 mm, Waters, Massachusetts, USA). The mobile phase was water with 0.05% formic acid (FA, Chem-Supply, Gillman, South Australia) and methanol with 0.05% FA in a gradient of 100:0–70:30, needle wash was MilliQ water at a flow rate of 0.4 mL/min, and an injection volume of 10 μL. Retention time was at 3.4 min. Standard concentrations produced linearity of R1 = 1. It achieved a lower limit of detection for RCA I at 2.7 ng/mL and detected concentrations up to 1.35 mg/mL. Data was analyzed by the Copley Inhaler Testing Data Analysis Software (Inhalytix, Nottingham, UK) WIBU, USP Ph. Eur. 2.918 (Copley Scientific Ltd., Nottingham, UK).

#### Laser Diffraction

The aerosol size distribution analysis of RCA1 was assessed using laser diffraction by SprayTec (Malvern, Worcestershire, UK), equipped with inhalation cell and USP induction port as the cascade impactor studies, connected to a vacuum pump to control the flow rate (15L/min). The aerosols were measured using a particle refractive index of 1.33, dispersant refractive index of 1.0 and data acquisition at 2.5 kHz using 300 mm lens. Aerosolized RCA I solution at 0.6 mg/mL in HBSS was loaded in a PARI LC® Sprint jet nebuliser connected to a PARI Turbo Boy S compressor. The collected data were averaged from 5–25 s and reported as a volume undersize D_10_ (10th percentile undersize value µm), D_50_ (50th percentile undersize value µm), and D_90_ (90th percentile undersize value µm).

#### Cell Culture

H441 cell line (HTB-174, an epithelial-like cell isolated from the lung of a male with papillary adenocarcinoma.) was obtained from the American Type Culture Collection (ATCC, Virginia, USA). The cells were cultured in RPMI-1640 media (Gibco, Massachusetts, USA) supplemented with 10% v/v foetal bovine serum (FBS, Gibco, Massachusetts, USA), sodium pyruvate and 2 mL L-glutamine (Gibco, Massachusetts, USA). Cells were grown in 75 cm^2^ flasks (Greiner BioOne, Dublin, Ireland) under humidified (95% RH, 5% CO2) atmosphere at 37°C. The media was exchanged every 2 to 3 days and the cells were passaged weekly when confluency was observed, following the ATCC recommended guidelines.

#### Integration of AIR Model with H441 ALI Model

H441 cells were maintained at the ALI using the protocol described by Wong *et al.*, which supports epithelial differentiation and tight-junction formation [19]. To culture H441 epithelial cells in an ALI model, confluent cells were seeded onto the apical chamber of Transwell polyester membrane inserts (0.33 cm^2^, 0.4 µM pore size, Corning Costar, New York, USA) at a density of 3 × 10^5^ cells/cm^2^ by adding 100 µL of cell suspension. The basolateral chamber received 600 µL of media. After 48 h of incubation, the apical chamber media was aspirated and basolateral chamber replaced with a differentiation media prepared with 1% Insulin-Transferrin-Selenium (Gibco, Massachusetts, USA), 200 nM dexamethasone, RPMI 1640 (Gibco, Massachusetts, USA), and 10% FBS. The basolateral chamber media was replaced every 2 days for 14 days prior to experimentation. Four Transwell inserts were then connected to the medial and lateral lower airway branches, as shown in Fig. 2A, and RCA I solutions (1.047 × 10^–11^ M—4.19 × 10^–9^ M concentrations) were nebulised using a PARI nebuliser into the AIR model for 2 min at a flow rate of 15 L/min. After deposition, the Transwell inserts were removed from the AIR model and transferred to a 24-well plate, where 600 µL of pre-warmed HBSS (37°C) was added to each well. The cells were incubated for 2 h at 37°C, 5% CO_2_, and 95% relative humidity. Transepithelial electrical resistance (TEER) [20], paracellular permeability assay (P_app_) were measured and RCA I transport quantified using electrochemical analysis [21, 22].

#### Cytotoxicity Assay of RCAI using the MTS Assay

To determine the toxic aerosol dose of RCA I (Vector Laboratories, California, USA), in a 2D liquid–liquid interface (LLI) model for comparison against the AIR model, a cytotoxicity assay was used to evaluate cell viability of H441 cells against a range of RCA I concentrations. H441 cells were seeded at 2.5 × 10^5^ cells/mL in 96-well plates (100 µL) and incubated for 24 h at 37°C, 5% CO_2_. Following a 24 h treatment with RCA I solution in HBSS (2.096 × 10^–17^ to 2.25 × 10^–8^ M) supernatant was removed, cells washed with phosphate buffered saline and incubated with MTS reagent (Promega, USA) at 37°C for 3 h and absorbance was read at 490 nm on a SpectraMax M2 (Molecular Devices, California, USA).

### Electrochemical Analysis of RCA I

#### Development of the Micro-analytical Device (MAD)

**Fig. 3:**
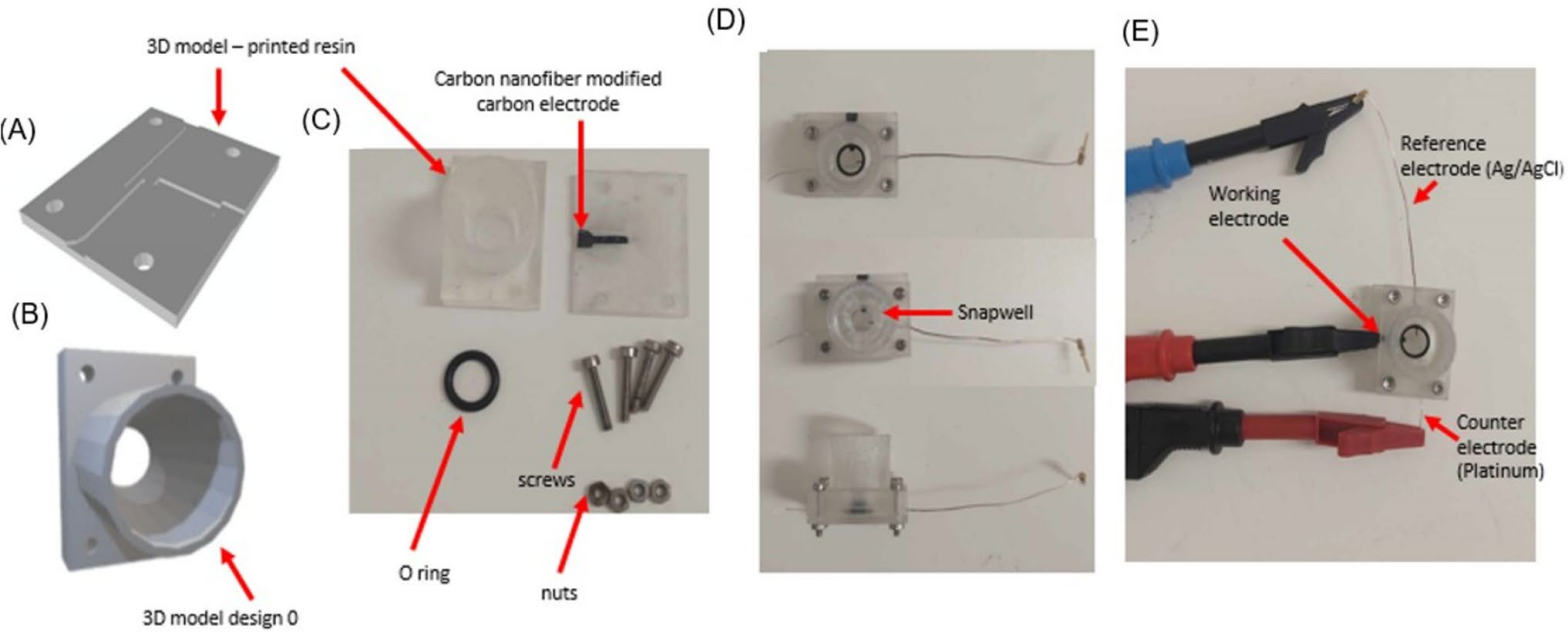
The structure of the MAD. (**A**, **B**) the 3D designs, (**C**) the materials for MAD assembly, (**D**) the assembled MAD, (E) the connections of the device to the potentiostat.

The two 3D-printed resin pieces (Fig. 3A, lower piece and 3B, upper piece) were assembled by connecting a sintered Ag/AgCl wire and a platinum wire, as illustrated in Fig. 3D. In Fig. 3A, the resin piece features two horizontal grooves: the left and right grooves. The Ag/AgCl electrode was positioned in the left groove, extending only to the midpoint of the chip, while the platinum wire was placed in the right groove, extending beyond the midpoint of the resin piece. To secure the pieces together, an O-ring was used, along with four screws and nuts (Fig. 3C). The three electrodes were then connected to the potentiostat (Metrohm Autolab, Utrecht, Netherlands) using crocodile clips, as shown in Fig. 3E.

#### Preparation of Carbon Nanofibers-modified Carbon (CNFs -C) Electrode

The resin chip of the MAD device was 3D-printed using FormLabs Clear Resin V4 (Massachusetts, USA) with the Form 3 + 3D printer, as shown in Fig. 3A and B. The printed chips were cured for 90 min at 60°C. Subsequently, the 3D-printed resin chips were covered with Scotch Magic™ tape, ensuring that only the center groove designated for the CNFs-C electrode remained exposed. The uncovered groove was then filled with carbon nanofiber paste [23].

To prepare the CNFs-C paste, carbon nanofibers (CNFs, Electron Microscopy Sciences, USA) were transferred into an Eppendorf tube, and 400 µL of conductive carbon cement (CCC, Electron Microscopy Sciences, Pennsylvania, USA) thinner was added, with additional CCC thinner used if the paste was too dry [24]. The mixture was sonicated for 10 min. Next, CCC was added to the Eppendorf tube at a 1:4 weight ratio (e.g., 50 mg CNFs to 200 mg CCC for 10 resin chips) under a fume hood and mixed until the paste became homogeneous. The prepared paste was applied to the designated slot for the working electrode (WE) on the 3D-printed resin chip using a fine spatula, ensuring a smooth surface (Fig. 3C). The CNFs-C electrode was then left to dry under the fume hood overnight before use.

#### Electrochemical Quantification of RCA1

RCAI sample collected from transport were added (150 µL) to the well of the MAD system for detection. Squarewave voltammetry (SWV) was used for quantification detections. SWV is a pulse voltammetric technique that measures both forward and reverse current, hence, can suppress the background current and is more sensitive than cyclic voltammetry or linear sweep voltammetry for quantification purposes [25]. SWV was performed at 0.126 V/s scan rate, at 25 Hz frequency and 0.02 V modulation amplitude, within 0.1 to 1.2 V potential range. It achieved a lower limit of detection for RCA I at 0.136 pg/mL and detected concentrations up to 1.36 mg/mL.

### Statistical Analysis

All results are presented as the mean ± standard deviation of three independent experiments. Statistical analysis was by one-way or two-way analysis of variance (ANOVA) with Tukey multiple comparisons using GraphPad Prism 9. Electrochemical analysis was caried out using the NOVA 2.1.4 program.

## Result and Discussion

Traditional *in vitro* assays for assessing the toxicity of inhaled aerosols typically involve the administration of liquid solutions or suspensions to two-dimensional (2D) cell monolayers. However, these methods fail to replicate key physiological aspects of the respiratory epithelium, such as ALI conditions, cellular differentiation, mucus secretion, and epithelial barrier function. Furthermore, they do not account for aerosolization, deposition, and transport dynamics. In this study, the AIR model was validated using an aerosolized fluorescent marker (Flu-Na), demonstrating reproducible deposition patterns and recovery within the expected range for impaction studies, as outlined by the United States Pharmacopeia. The integration of ALI cultures with the AIR model provided a physiologically relevant platform for evaluating inhalation toxicity, improving upon conventional cytotoxicity assays.

### Cytotoxicity of RCA I in 2D Cellular Model

**Fig. 4:**
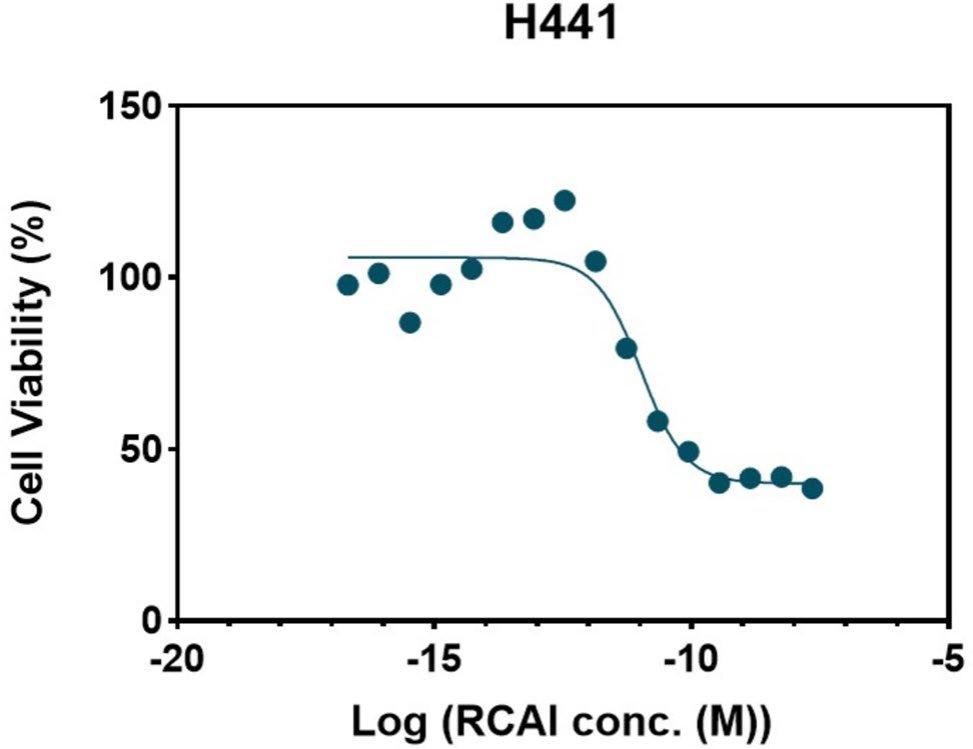
The cytotoxicity profiles of H441 treated with RCA I at different concentrations. mean ± STD of 3 independent experiments.

Cytotoxicity assay on a range of RCA I concentrations on H441 cells in a LLI model found that the inhibitory concentration IC (amount of drug required to inhibit the metabolic activity of cells by 10, 50 and 80%) was IC10 1.16 × 10^–12^, IC50 1.047 × 10^–11^, and IC80 4.19 × 10^–11^ M, respectively (Fig. 4). These concentrations were used to define the expected cytotoxicity profile under submerged conditions, serving as a benchmark for comparison with the response seen in the AIR model.

### *In vitro* Deposition Profile of RCA I

**Fig. 5:**
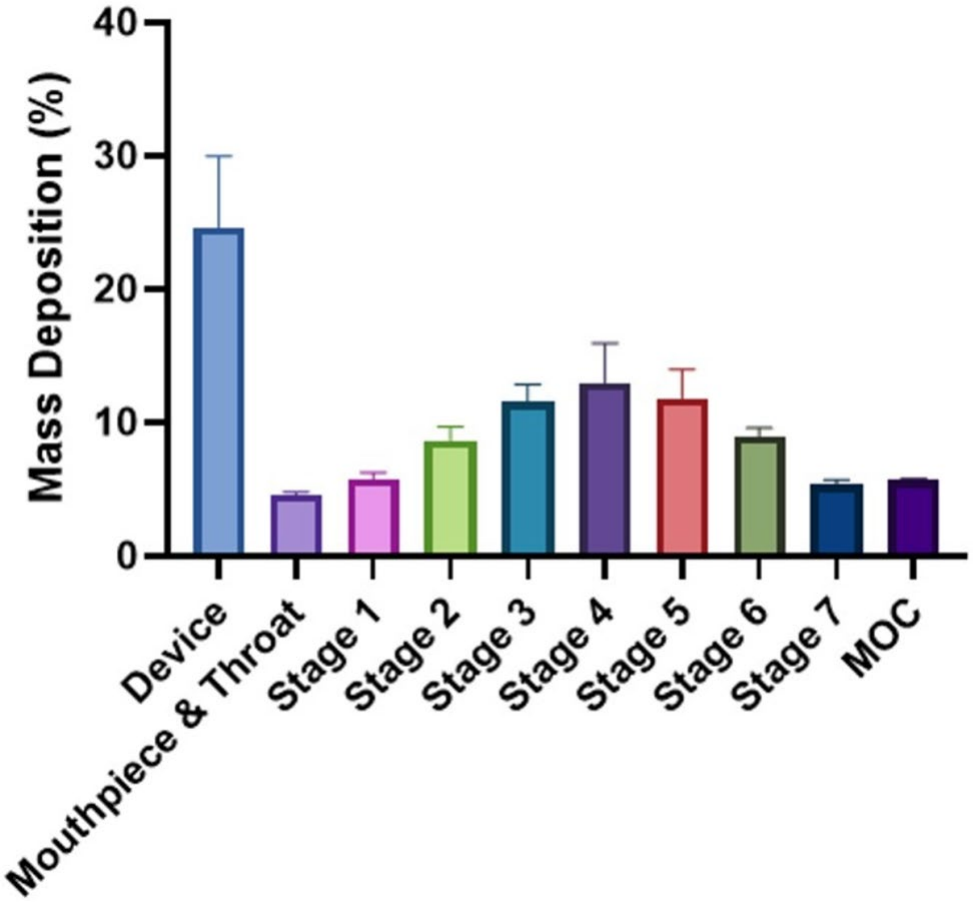
*In vitro* deposition pattern of RCAI in the NGI. RCAI solution (4 mL, 0.6 mg/mL) in HBSS was aerosolized by PARI LC® Sprint jet nebulizer at a flow rate of 15 L/min (n = 3, mean ± SD).

Figure 5 shows the *in vitro* aerosol performance of RCA I solution in HBSS. The deposited mass of RCA I is presented as a percentage of the total mass collected from all components of the NGI. The majority of the RCA I remained in the nebuliser, with the highest deposition found at stage 4 (3.30 µm cut-off) of the NGI [26]. This is reflected by the mass median aerodynamic diameter (MMAD) of 3.78 ± 0.3 µM. A high percentage of RCA I was deposited at the micro-orifice collector (MOC, 0.98 µm cut-off) which indicated the PARI Jet Nebuliser was generating nanodroplets, which is further supported by the DV_10_ values of RCA I at 0.70 ± 0.07 µm. The fine particle fraction (FPF, % of total drug ≤ 5 µm to the delivered dose) of RCA I was 56.84 ± 3.3%, and delivered dose was greater than 70% of the total dose, suggesting efficient deposition in the lower respiratory tract. These aerosol characteristics are indicative of deposition within the distal respiratory airways. This aligns with the physiological relevance of the model, as H441 cells recapitulate key features of the distal airway epithelium [11].

### Characterization of Aerosol Deposition in the AIR Model using Flu-NA Aerosols

As shown in Fig. 2B, the transparent components of the AIR model allow for visualization of the deposition pattern of the aerosols in the airways. The heterogenous deposition of aerosols inside the complex geometry of the upper airway, with the maximum deposition in the lower part of the oral cavity, epiglottis, as well as the deposition into the bifurcations of the lower airways. The deposition pattern is consistent with the NGI data. In both the AIR model and NGI, the majority of the delivered RCAI was deposited in the lower airways and chamber, corresponding to NGI stages 3 – MOC (5.39 µm – 0.98 µm). Less than 10% of the deposited RCAI was recovered in the upper airways of the AIR model and the throat region of the NGI.

**Fig. 6:**
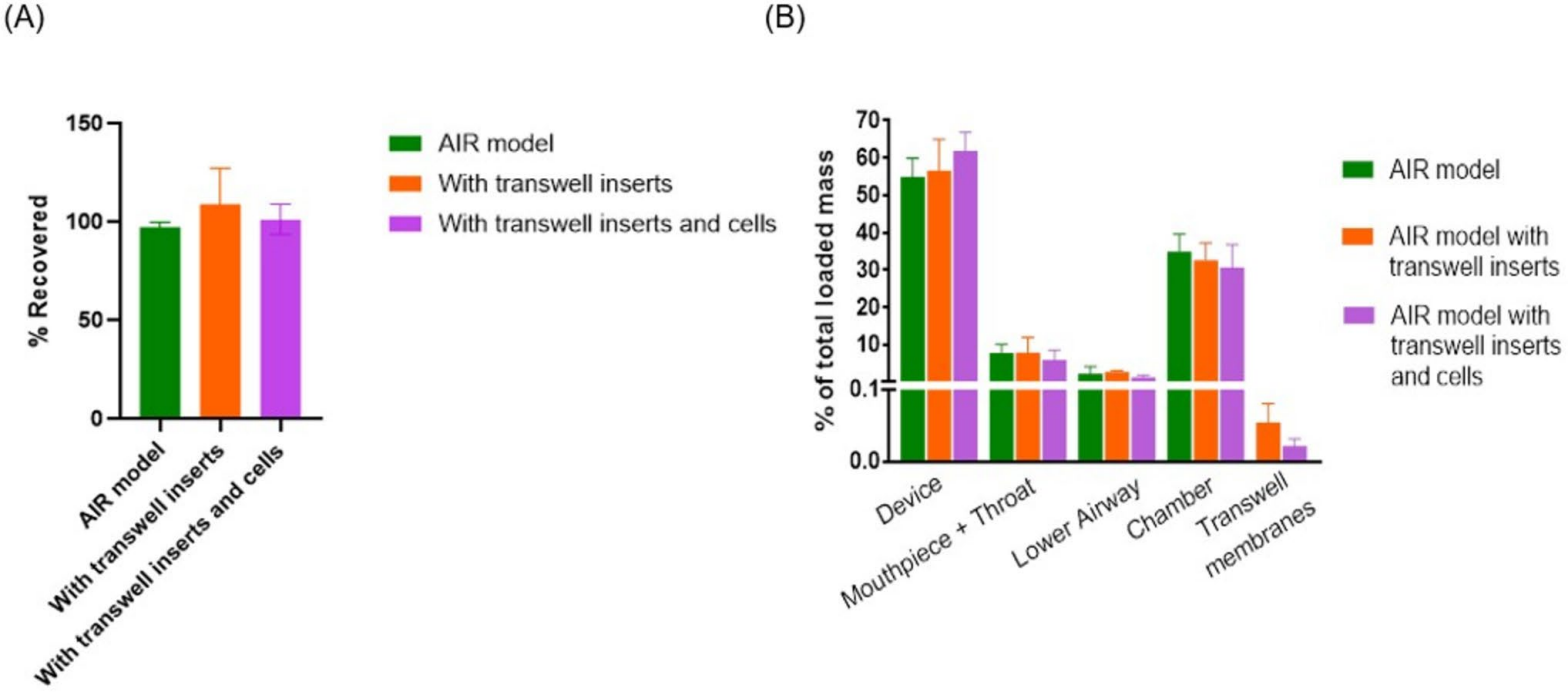
Flu-Na mass deposition in AIR model components with/without Transwell inserts (ALI H441 cells present/absent). (**A**) % of recovered Flu-NA aerosol mass within ± 10% of the total Flu-Na mass (**B**) Mass deposition of Flu-Na in AIR model components measured by fluorescence at 486 and 583 nm. N = 3, mean ± STD, ns (p > 0.05).

The deposited mass was calculated as a percentage of the total mass collected from the AIR model and shown in Fig. 6. Most of the Flu-Na mass was deposited in the chamber, with a similar percentage deposited between the mouthpiece, throat and lower airway. The Transwell membranes were shown to have the lowest deposition of Flu-Na. Importantly, comparing the AIR model alone, the AIR model with empty Transwell inserts, and the AIR model with cell-containing inserts showed no significant differences in deposition across any part of the system (0.11 ≤ p ≤ 0.71). This confirms that adding inserts, with or without cells, does not influence aerosol distribution and supports the robustness and reproducibility of the model.

### Toxicity of RCA I in AIR Model

**Fig. 7:**
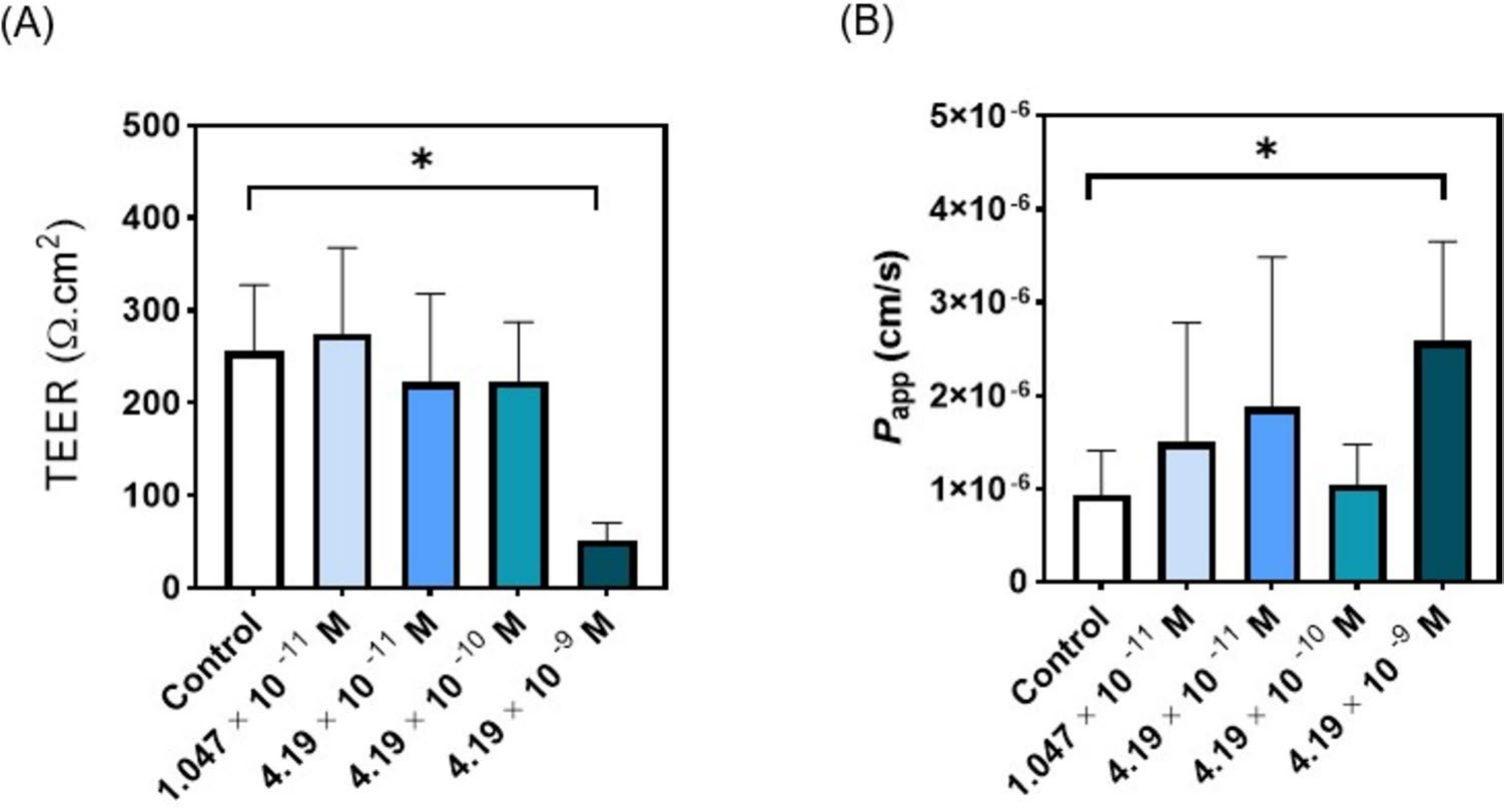
The effect of RCA I aerosol delivered to the Transwell inserts with H441 attached to the lower airway of the AIR model. (**A**) TEER and (**B**) Papp results for H441. mean ± STD. *p < 0.05; one-way ANOVA with Tukey’s multiple comparison test was performed.

Four different concentrations of RCA I solutions were nebulised to determine the toxic dose on H441 cells (Fig. 7) using the AIR model. The initial two concentrations were chosen based on MTS results, with two more concentrations chosen by increasing one order of magnitude to attain a significant toxic dose. The highest concentration (4.19 × 10^–9^ M) of RCA I tested resulted in a significant drop in the TEER, shown in Fig. 7A. The P_app_ results in Fig. 7B for different concentrations showed a significant difference between the control cells and the cells exposed to the 4.19 × 10^–9^ M RCA I aerosol, however, no significant difference was observed between the control cells and the cells exposed to the other 3 concentrations. The observations in TEER and P_app_ assays together showed a toxic effect when 4.19 × 10^–9^ M of RCA I was aerosolized onto H441 cells.

The toxic dose in the AIR model is approximately three orders of magnitude higher than the IC50 values compared to traditional 2D assays, as determined by TEER and P_app_ measurements. These findings highlight the limitations of metabolic assays such as MTS, which do not capture barrier integrity, which is a primary determinant of airways epithelial function *in vivo* [19]. Notably, exposure to the highest RCA I concentration (4.19 × 10⁻⁹ M) resulted in a significant decrease in TEER and an increase in P_app_, indicating epithelial barrier disruption.

**Fig. 8:**
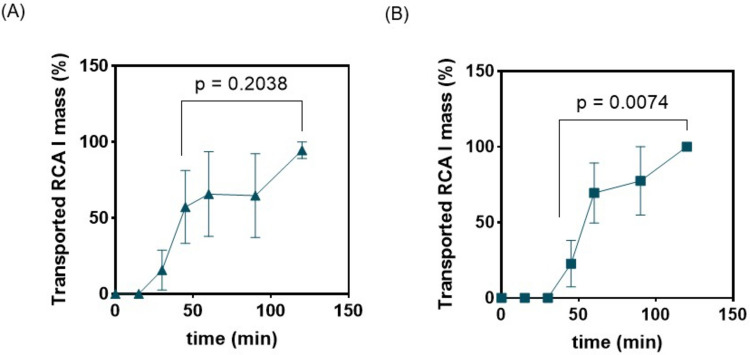
Time course of the RCA I aerosol transport across the H441 cells at (**A**) toxic (4.19 × 10^–9^ M) and (**B**) non-toxic (1.047 × 10^–11^ M) doses. N = 3, mean ± STD.

After determining the toxic and non-toxic doses of RCA I aerosolized solution across the ALI models of H441, the RCA I transport was evaluated using the micro-analytical device based on the EC SWV technique. Transport profiles of RCA I at toxic dose showed a significantly higher rate of mass flux compared to non-toxic dose, as most of the transport occurred in the first 45 min for the toxic dose, which then plateaued until the end of the 2 h experiment (Fig. 8). This suggests the impaired cellular barrier at the toxic RCA I dose, which facilitates rapid toxin penetration, underscoring the necessity of ALI-based models for accurately assessing inhalation toxicity.

In summary, the AIR model effectively assesses lung physiology and inhalation exposure, allowing for precise characterization of aerosol interactions with respiratory epithelium. Unlike traditional 2D assays, the AIR model captures airway geometry, airflow, real-time monitoring, and epithelial barrier function, allowing exposure patterns and injury responses that better reflect *in vivo* inhalation. The much higher dose required to compromise epithelial integrity in the AIR model highlights the need for physiologically relevant *in vitro* platforms for inhalation toxicology.

## Limitations

While the AIR model provides a physiologically relevant *in vitro* platform for studying aerosolized toxins, several limitations should be acknowledged. First, the AIR model does not fully replicate the dynamic nature of *in vivo* lung physiology, including mucociliary clearance, immune cell interactions, and pulmonary surfactant effects, which may influence toxin retention and transport [27]. Secondly, while a constant vacuum flow of 15 L/min was used in the AIR model to align with the NGI testing, inspiratory flow rate for adults may be higher, so it may be useful to test at 30 L/min as well [28]. Additionally, temperature and humidity within both the external and internal environments of the AIR model were monitored at 23 ºC and 45–55% relative humidity but not controlled. Variations in these parameters may alter aerosol behaviour, including droplet evaporation and deposition patterns [29]. Controlling temperature and humidity will be an important future step to enhance physiological relevance. It is also worth noting that the model’s unidirectional, steady-state flow does not capture the biphasic, cyclic pattern of human breathing.. As such, expiratory losses of ultrafine particles are not captured, which may lead to overestimation of distal lung deposition and underestimation of airway retention. Future iterations of the model would benefit from incorporating physiologically relevant, dynamic breathing patterns to better simulate transitional flow effects. This study showed no significant differences in Flu-Na deposition between the AIR model with Transwell inserts, inserts seeded with cells, or no inserts at all, supporting the robustness of the system. We acknowledge, however, that the 12 open branches without inserts may introduce slight airflow asymmetry. Future versions will include pore-size–matched membrane filters at all open outlets to provide uniform resistance and improve flow symmetry. Additionally, the study relied on a single cell line (H441) cultured at an ALI interface, which, while useful for evaluating epithelial barrier function, does not account for the complex cellular heterogeneity of the human respiratory tract. Another limitation lies in the aerosol deposition methodology; while the NGI provides insights into aerodynamic properties, *in vivo* validation through animal models or clinical deposition studies would strengthen the translatability of these findings. Finally, while the cytotoxicity assays demonstrated clear dose-dependent toxicity, additional endpoints, such as inflammatory cytokine release and oxidative stress markers, should be explored to provide a more comprehensive assessment of toxin-induced lung injury. Addressing these limitations in future studies will enhance the robustness and clinical relevance of the AIR model for inhalation toxicology and aerosol-based drug delivery research.

## Conclusions

Overall, the AIR model represents a robust and reproducible *in vitro* system for assessing aerosolized agents, offering a more physiologically relevant alternative to traditional methods. This platform has the potential to enhance the evaluation of inhalation toxicology and drug delivery, ultimately contributing to improved risk assessment and therapeutic development in respiratory research.
